# Home-Based SCONE^TM^ Therapy Improves Symptoms of Neurogenic Bladder

**DOI:** 10.1089/neur.2020.0061

**Published:** 2021-03-18

**Authors:** Parag Gad, Hui Zhong, V. Reggie Edgerton, Evgeniy Kreydin

**Affiliations:** ^1^ Department of Neurobiology, Keck School of Medicine, University of California, Los Angeles, Los Angeles, California, USA.; ^2^ Rancho Research Institute, Rancho Los Amigos National Rehabilitation Center, Downey, California, USA.; ^3^ SpineX, Inc., Los Angeles, California, USA.; ^4^ Department of Neurosurgery, Keck School of Medicine, University of California, Los Angeles, Los Angeles, California, USA.; ^5^ Brain Research Institute, Keck School of Medicine, University of California, Los Angeles, Los Angeles, California, USA.; ^6^ Institut Guttmann, Hospital de Neurorehabilitació, Institut Universitari adscrit a la Universitat Autònoma de Barcelona, Barcelona, Badalona, Spain.; ^7^ Department of Urology, University of Southern California, Los Angeles, California, USA.

**Keywords:** non-invasive spinal cord stimulation, neurogenic bladder, overactive bladder, stroke, urodynamics

## Abstract

A wide range of dysfunction can occur after a stroke including symptoms such as urinary urgency, frequency, and urge incontinence. The Spinal Cord Neuromodulator (SCONE^TM^) reactivates and retrains spinal neural networks. The present case study introduces initial evidence that home-based, self-administered SCONE therapy may be a safe and effective method of delivering this neuromodulation modality and may have the ability to minimize clinic visits, which is especially salient in today's public health environment.

## Introduction

Each component of the nervous system plays a role in the control of the lower urinary tract (LUT) with the brain classically thought to exert an inhibitory influence.^1^ In the case of cerebral stroke, LUT dysfunction occurs because the input to the LUT from the brain is either completely lost or disrupted. A wide range of dysfunction can occur after a stroke, but patients most commonly develop storage symptoms such as urinary urgency, frequency, and urge incontinence characterized by detrusor overactivity and resulting in a significant impact on health and quality of life.^2^ Several current therapies that target the LUT have adverse effects that limit their acceptability in the stroke population: for example, anticholinergics may have an adverse effect on post-stroke cognitive function and botulinum toxin may lead to urinary retention in the most common type of stroke victim—the elderly male.^3^ Because stroke is one of the most prevalent causes of long-term disability in the world and often affects the already-frail members of the population, there exists a significant need for therapies that are safe, well-tolerated, easily administered, and effective. As a therapy aimed at restoring normal neural function, non-invasive self-delivered neuromodulation may meet these criteria.

Recently, we developed a non-invasive Spinal Cord Neuromodulator (SCONE^TM^) to reactivate and retrain spinal neural networks with the goal of restoring function in patients with neurological disease, such as spinal cord injury, stroke, and multiple sclerosis.^4^ This approach consists of using specific neuromodulatory parameters that transform dysfunctional neural networks of the spinal cord to functionally physiological states. We hypothesized that SCONE engages the automaticity and the feed-forward^5^ features intrinsic to spinal neural networks to enable recovery of voluntary control of continence^4^ and voiding.^6,7^ In addition, unlike existing neuromodulation modalities (sacral and tibial) this approach has the unique feature of delivering stimulation to the spinal cord directly without relying on peripheral intervention. Traditionally, non-invasive delivery of an electrical stimulus to deep nervous structures, such as the spinal cord, has been a challenge due to cutaneous discomfort caused by high current levels required to reach these structures. SCONE circumvents this issue by delivering a combination of multiple frequencies that render high-amplitude waveforms comfortable even in fully able-bodied individuals.^8^

**FIG. 1.**
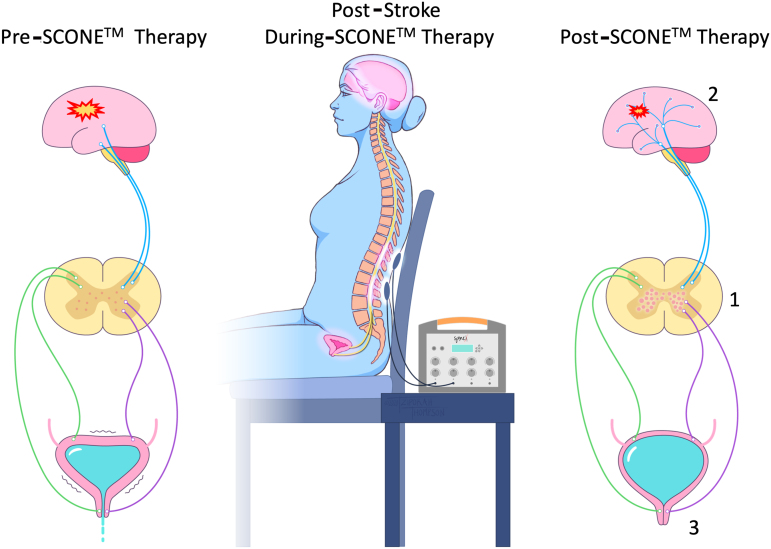
Schematic representation of a stroke patient receiving Spinal Cord Neuromodulator (SCONE^TM^) therapy. Note changes occur at multiple levels: 1) activation of local lumbosacral spinal neural networks, 2) changes in sensation and control at the cortical level, and 3) increase in voluntary control of urethra and reduction in urge and urge urinary incontinence.

## Case Study

Our earlier pilot study provided promising preliminary data that SCONE administered in the clinic setting may have a significant beneficial effect on stroke-related LUT dysfunction.^4^ In the present case study, we sought to explore the feasibility, safety, and efficacy of SCONE when it is self-administered in the home environment.

C.H.P. is a 49-year-old male with a history of left basal ganglia hemorrhagic cerebrovascular accident that occurred 6 years prior to study initiation. The patient developed right hemiparesis, speech impediment, and storage LUT symptoms as a result of this event. Lower urinary tract symptoms (LUTS) consisted of severe urinary frequency (up to 10–12 times a day), nocturia (up to 6–8 times per night), urinary urgency, and occasional urge incontinence. Initial urodynamic evaluation revealed detrusor overactivity with associated incontinence at a low volume (68 mL). The detrusor overactivity contraction was at 53 cm H_2_O and a flow of 14 mL/sec, suggesting that no bladder outlet obstruction was present. A trial of anticholinergic therapy for 6 months was not beneficial. An intravesical injection of onabotulinumtoxinA 100 units was attempted but this resulted in incomplete bladder emptying and exacerbation of his symptoms. Approximately 1 year after the onabotulinumtoxinA injection, he completed 12 sessions of percutaneous tibial nerve stimulation (PTNS). This therapy resulted in mild improvement of his symptoms: his nocturia decreased to 5–6 episodes a night and he noted a subjective improvement in urgency. However, most of his LUTS returned to baseline levels within a few weeks of completing PTNS.

Between September 2018 and November 2018, the patient completed a course of SCONE therapy in the clinic (3 times per week for 8 weeks), after which he reported a reduction in neurogenic bladder symptom score (NBSS) by 8 points (23 to 15 points, *p* < 0.05), a reduction in daytime frequency (10.35 to 7.5 times per day, *p* < 0.05), and a reduction in nocturia (6.4 to 4.25 times per night, *p* < 0.05), as reported earlier.^4^ There were no changes in bladder capacity during urodynamic studies compared with his baseline. However, he reported improvement in controlling the sudden urge to void, that is, the ability to hold urine at capacity prior to voluntarily initiating voiding. This observation was validated during the urodynamic study by a longer time window between reaching bladder capacity and involuntary initiation of voiding. On follow-up, the patient sustained the improved level of functionality for 3–4 months. After this period, LUTS returned to the patient's pre-stimulation state.

Between August 2020 and October 2020, the patient completed a course of SCONE therapy at home (5 times a week for 12 weeks), after which he reported a reduction in NBSS by 5 points (19 to 14 points, *p* < 0.05), a reduction in daytime frequency (9.25 to 7.75 times per day, *p* < 0.05), and a reduction in night-time frequency (2.5 to 1.75 times per night, *p* < 0.05). A small increase in average voided volume (from 98 to 112 mL) was observed at the end of 8 weeks. In addition, he also reported a reduction in average urge prior to voiding (1.08 to 0.55, *p <* 0.05), a reduction in frequency of occurrence of urge levels of 2 (on a scale of 0 [no urge] to 4 [about to leak]) or greater (3.75 to 2, *p* < 0.05), and an increase in frequency of voiding without any urge (4 to 7.5, *p* < 0.05). The changes in behavior were not due to a decrease in fluid consumed as recorded from the 4-day average voiding diary: 1788 mL versus 1837 mL. The intensity of SCONE delivered during therapy in the clinic and at home ranged between 60 and 80 mA. The patient experienced no discomfort or pain due to the stimulation. No adverse events were reported and the degree in symptom improvement was similar or better than what the patient experienced during Transcutaneous Electrical Spinal Cord Neuromodulator (TESCoN) therapy delivered in the clinic.

Subsequently, during the next 3 months of follow-up without SCONE therapy, the patient reported a sustained level of functionality with an NBSS score of 12, an average urge prior of voiding of 0.3, a minimal change in frequency of occurrence of urge levels of 2 or greater (once per day), with no change in nocturia frequency (2.3) and frequency of voiding without any urge (6.3). Due to impairment in hand function, the electrodes were placed on his back by his primary caregiver. Although self-placement of electrodes is a potential limiting factor to this approach, the benefits observed outweigh the costs associated.

Oral medications and botulinum toxin improve LUTS by diminishing bladder contractility and bladder sensation.^9^ On the other hand, the premise of neuromodulation is to deliver a low-level electrical stimulation to change the excitability of neural networks involved in LUT control. Thus, restoration of function and correction of dysfunction are the basis of neuromodulation approaches to the LUT. We hypothesize that spinal cord stimulation modulates the interneuronal networks and transforms both afferent and efferent networks into more functional states. Neuromodulation alters the responsiveness of spinal networks to bladder filling and emptying and increases the conscious awareness of these states. After chronic SCONE therapy, patients have reported improved bladder sensation and decreased urinary urgency.^4^ In addition, the case patient in the present study was capable of sustaining the level of functionality for several months post-therapy. Together, these findings suggest that parts of the central nervous system (CNS) responsible for conscious sensation in the brain may have been re-engaged along with re-activation and/or re-training of local spinal centers controlling the LUT, reflecting a highly significant level of functional neural plasticity.

## Conclusion

The present case study introduces initial evidence that home-based, self-administered SCONE therapy may be a safe and effective method of delivering this neuromodulation modality. The ability to minimize clinic visits is especially salient in today's public health environment. We acknowledge that these findings cannot be interpreted to imply results from a large population of patients. However, current and previous results provide a significant level of confidence that SCONE has the potential to significantly ameliorate symptoms of autonomic dysfunction such as neurogenic bladder^4^ and motor dysfunction such as paralysis.^10^

## Abbreviations Used

CNS: central nervous system
LBNS: neurogenic bladder symptom score
LUT: lower urinary tract
LUTS: lower urinary tract symptoms
PTNS: percutaneous tibial nerve stimulation
SCONE: Spinal Cord Neuromodulator
TESCoN: Transcutaneous Electrical Spinal Cord Neuromodulator
